# Kallmann Syndrome Due to Heterozygous Mutation in *SOX10* Coexisting With Waardenburg Syndrome Type II: Case Report and Review of Literature

**DOI:** 10.3389/fendo.2020.592831

**Published:** 2021-02-01

**Authors:** Kan Chen, Haoyu Wang, Yaxin Lai

**Affiliations:** Liaoning Provincial Key Laboratory of Endocrine Diseases, Department of Endocrinology and Metabolism, Institute of Endocrinology, The First Hospital, China Medical University, Shenyang, China

**Keywords:** Kallmann syndrome, Waardenburg syndrome type Ⅱ, *SOX10* mutations, sensorineural deafness, hypogonadotropic hypogonadism, co-occurrence

## Abstract

**Introduction:**

Kallmann syndrome (KS) is idiopathic hypogonadotropic hypogonadism with olfactory loss or decline. Waardenburg syndrome type II (WS2) is a clinically and genetically heterogeneous disease, characterized by congenital sensorineural deafness and abnormal pigmentation of the iris, hair, and skin. Recently, mutations in the well-known WS pathogenic gene *SOX10* have been found in some KS patients with deafness, but whether *SOX10* is a co-pathogenic gene of KS and WS remains uncertain. Here, we report a rare case of KS and WS2 co-occurrence due to *SOX10* mutations.

**Methods:**

Detailed histories were collected through questionnaires and physical examination. Blood samples of the patient and his family members were collected after obtaining informed consents. Suspected mutations were amplified and verified by Sanger sequencing after the next generation sequencing of related genes. The raw sequence data were compared to the known gene sequence data in publicly available sequence data bases using Burrows-Wheeler Aligner software (BWA, 0.7.12-r1039).

**Results:**

A 28-year-old male patient sought treatment for hypogonadism and the absence of secondary sexual characteristics. In addition, he showed signs of obesity, hyposmia, sensorineural hearing loss, and blue iris. Magnetic resonance imaging (MRI) of the olfactory bulb showed small bilateral olfactory bulbs and tracts and diaphragma cerebri. MRI of the pituitary gland revealed a flat pituitary gland in the sella. Laboratory examination demonstrated hypogonadotropic hypogonadism, pituitary hypothyroidism, subclinical hypothyroidism, and the presence of insulin resistance with normal blood glucose levels. Sequencing of the SOX10 gene showed a 20 bp insertion in between coding bases 1,179 and 1,180 (c.1179_1180insACTATGGCTCAGCCTTCCCC). This results in a frame-shifting mutation of the 394th amino acid serine in exon4 with the resulting the amino acid sequence of the protein predicted to be TMAQPSP PSPAPSLTTL TISPQDPIMA TRARPLASTR PSPIWGPRSG PSTRPSLTPA PQGPSPTAPH TGSSQYIRHC PGPKGGPVAT TPRPAPAPSL CALFLAHLRP GGGSGGG*.

**Conclusion:**

*SOX10* plays an important role in some critical stages of neural crest cell development and *SOX10* mutation may be a common pathogenic factor for both KS and WS. Therefore, *SOX10* mutation analysis should be considered for KS patients with combined WS clinical manifestations, especially deafness.

## Introduction

Kallmann syndrome (KS) is a clinically and genetically heterogeneous disease with multiple genetic patterns of inheritance including autosomal dominant (65%), autosomal recessive (25%), and X-linked recessive (10%). The etiology and pathogenesis of KS is a disturbance in the common neural migration pathways of gonadotropin-releasing hormone (GnRH) neurons and olfactory neurons early in embryonic development, and the main clinical manifestations are hypogonadism and anosmia (1). At present, more than 20 pathogenic genes have been found to be associated with KS, of which six are relatively common (*KAL1, FGFR1, PROKR2, PROK2, CHD7, FGF8*) (2–6). However, only about 30% of the etiology and pathogenesis can be explained by the reported genetic mutations currently and about 70% remains unknown. Although numerous KS cases have been reported in the literature, co-occurrence of KS and Waardenburg syndrome type II (WS2) has only been reported twice (7, 8).

KS is often associated with other developmental deficiencies, such as hearing impairment that is found in about 5% of KS patients. In 2013, Pingault et al. (9) found that approximately 38% of patients with KS and deafness have *SOX10* mutations. It is well known that *SOX10* mutations are one of the causative gene mutations associated with WS, found in about 15% of WS2 patients and 45% of WS4 patients. The characteristic clinical manifestations of WS2 are congenital sensorineural deafness and abnormal pigmentation of the iris, hair, and skin. WS4 is a combination of clinical manifestations of WS2 and Hirschsprung disease (10, 11). The clinical manifestations of WS3 and WS1 are similar and their striking features are accompanied by abnormal development of the facial or upper limb musculoskeletal structures. However, the genetic link between KS and WS has not been established, and the clinical manifestations caused by *SOX10* mutations need to be fully elaborated.

In this case study, we identified a novel compound heterozygous mutation in the *SOX10* gene (c.1179_1180insACTATGGCTCAGCCTTCCCC, p.Ser394fs) in a Chinese patient with both KS and WS2.

## Methods and Result

### Case Presentation

The 28-year-old male patient was born to non-consanguineous Chinese parents after a normal pregnancy. He had bilateral sensory deafness and blue irises at the time of birth. His mother noticed his lack of sense of smell in childhood. His mental development was normal. He had no Hirschsprung disease or episodes of constipation. The abnormality of microphallus was first noticed by his family 10 years ago, but he had not been medically examined for it, and no improvement was seen after taking traditional Chinese medicine. Therefore, he was first referred to the First Affiliated Hospital of China Medical University for evaluation of the short penis at the age of 28. Both the patient’s mother and brother were healthy. However, his father had died.

### Physical Examination

**Figure 1:**
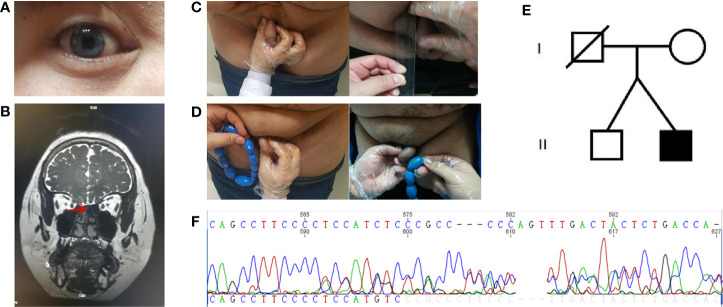
Clinical characteristics and gene sequencing results of the patient and his family members. **(A)** Depigmentation of the irides. **(B)** Magnetic resonance imaging of the olfactory bulb. **(C)** Comparison of stretched penis length before and after treatment. **(D)** Comparison of testicular volume before and after treatment. **(E)** The proband’s family pedigree. **(F)** The *de novo* mutation in *SOX10* gene (c.1179_1180insACTATGGCTCAGCCTTCCCC, p.Ser394fs).

The patient showed the following features: height, 180 cm; weight, 103 kg; body mass index (BMI), 31.97 kg/m2; body temperature, 36.5°C; blood pressure, 120/76 mmHg; pulse rate, 74/min; respirations, 18/min; blue irises (**Figure 1A**); lack of facial hair; white forehead; lack of acne; lack of moon face; lack of underarm hair; lack of thyroid swelling; enlarged breasts and mild tenderness; palpable breast nodules; lack of galactorrhea; sparse pubic hair; a stretched penis length of 3 cm (**Figure 1B**); a small bilateral testicular volume of 6.0 ml (**Figure 1C**).

### Assessment

Scrotal ultrasound showed a reduced size of the testes and the presence of microcalcification in them. His sense of smell was tested formally (Toyota-Takagi olfactometer test) and hyposmia was discovered. Audiologic examination demonstrated a sensorineural hearing loss. Magnetic resonance imaging (MRI) of the olfactory bulb showed small bilateral olfactory bulbs and tracts, and diaphragma cerebri (**Figure 1D**). Bone mineral density analysis revealed decreased bone density in the hip joint and lumbar vertebrae. MRI of the pituitary gland revealed no enlargement of the sella turcica or collapse of the saddle bottom but a flat pituitary gland in the sella. Ultrasound of breasts and bilateral axillary lymph nodes revealed bilateral male breast development. Thyroid gland and bilateral cervical lymph node ultrasound demonstrated a single hypoechoic nodule in the right thyroid lobe (TI-RADS 3+level). Liver ultrasound showed a fatty liver. No definite abnormality was found on computed tomography of the lungs and adrenal glands. Chromosomal analysis revealed a 46, XY karyotype. Laboratory examination demonstrated hypogonadotropic hypogonadism, pituitary hypothyroidism, subclinical hypothyroidism, and the presence of insulin resistance with normal blood glucose levels (**Table 1**).

**Table 1:** Clinical and laboratory data of the patient at diagnosis.

		At diagnosis		Follow up (3 month)		Normal range	
Weight(kg)		103		100		–	
Hight(cm)		180		180		–	
T(nmol/L)		<0.69		11.47		9.08–55.23	
FT(pmol/L)		7.36		65.49		55.05–183.50	
LH(mIU/L)		0.71		–		0.8–7.6	
FSH(mIU/L)		0.97		–		0.7–11.1	
ACTH(pg/mL) (8:00 a.m.)		14.66		–		7.20–63.30	
COR(nmol/L) (8:00 a.m.)		504.4		–		171–536	
TSH(mIU/L)		6.53		4.64		0.35–4.94	
FT4(pmol/L)		12.00		13.04		9.01–19.05	
FT3(pmol/L)		4.27		3.91		2.63–5.7	
	Fasting		30 min later	60 min later		120 min later	180 min later
PG(mmol/L)	5.51		6.37	4.65		5.89	5.28
INS(mIU/L)	30.87		243.20	135.70		176.1	43.78
CP(pmol/L)	1554.00		7683.70	4145.90		6888.70	1921.40

T, testosterone; FT, free testosterone; LH, luteinizing hormone; FSH, follicle-stimulating hormone; FSH, follicle-stimulating hormone; ACTH, adrenocorticotropic hormone; COR, cortisol; TSH, thyrotropin-releasing hormone; fT4, free thyroxine; fT3, free triiodothyronine; PG, plasma glucose; INS, serum insulin; CP, serum C peptide.

### Genetic Testing

Blood samples were collected from the patient and his family members (mother and brother). Genomic DNA was extracted using a blood extraction kit (Tian Jing Biochemical Technology Beijing, Ltd.). Roche NimbleGen SeqCap EZ Choice XL Library was used for exon trapping (including 4,132 genes). Illumina NextSeq500 was used for high-throughput sequencing. Burrows-Wheeler Aligner software (BWA, 0.7.12-r1039) was applied to align the sequencing data to the human genome. ANNOVAR was used to annotate the genetic variants detected. Emphasis was laid on the analysis of the known genes involved in KS and WS pathology. The results revealed that a large fragment of base insertion at the site 1,179–1,180 of *SOX10* (c.1179_1180insACTATGGCTCAGCCTTCCCC, p.Ser394fs) resulted in a heterozygous mutation in exon4 (**Figure 1E**). The frame-shifting mutation of the 394th amino acid serine resulted in a series of code changes downstream. Predicted by MutationTaster (disease-causing, score=1) software, it is related to the disease. After querying in databases such as The Single Nucleotide Polymorphism Database, The Human Gene Mutation Database, 1000 Genomes Project, ClinVar, Exome Sequencing Project v.6500 database, and related literature, it was found that this mutation had not been reported as a new mutation. When the coding region of exon4 of *SOX10* was resequenced in the samples of the patient’s mother and brother, the insertion was not detected (**Figure 1F**).

### Therapeutic Intervention and Follow-Up

For hypogonadism, 2,000 U human chorionic gonadotropin (hCG) was intramuscularly injected twice a week, and testosterone and free testosterone levels were re-examined periodically at outpatient visits. Additionally, 75 U of human menopausal gonadotropin (hMG) was added to the patient’s medications 3 months later. For subclinical hypothyroidism, regular monitoring of thyroid function was carried out. Subsequently, the underarm and pubic hair of the patient showed significant growth. The length of the penis increased to 5 cm (**Figure 1B**), testicular volume increased to 8 ml (**Figure 1C**), and his testosterone levels increased as well and reached the normal range (**Table 1**). Thyroid function also returned to normal (**Table 1**).

## Discussion

Herein, we report a case of a man with KS and WS2, who carried a novel insertion of a 20 bp fragment into the coding sequence of the exon4 of the *SOX10* gene. Our patient exhibited hypogonadotropic hypogonadism and anosmia, which are characteristics of KS. Interestingly, he also manifested characteristics of WS2 such as bilateral sensorineural deafness and abnormal pigmentation (blue irises, white forehead hair). To date, 18 patients with KS have been identified to have the *SOX10* mutation, of which 15 patients had unilateral or bilateral sensorineural deafness, accounting for 83% of the KS patients with *SOX10* mutation. In addition, five of the 18 patients demonstrated abnormal pigmentation and other typical clinical manifestations of WS. However, Hirschsprung disease was not described in any of the 18 patients (**Table 2**). Our report and previous findings indicate the importance of *SOX10* haploinsufficiency as one of the genetic causes of KS with WS-characteristic clinical features. It is well known that *SOX10* is an important pathogenic gene associated with WS2 and WS4, and KS with deafness is similar to WS with anosmia in many clinical manifestations, such as perceptive nervous deafness, abnormal pigmentation, and hyposmia. Therefore, it can be speculated that there is a certain association between the two diseases.

**Table 2:** Phenotypes of the KS Individuals Carrying SOX10 Mutations.

Case	Age	Gender	Occurrence	FSHBasalPeak(IU/L)	LHBasalPeak(IU/L)	E2(pg/ml)	T(ng/ml)	SpontaneousPuberty	Sense of Smell	Hearing	Pigmentation Abnormalities	Other Clinical Signs	DNA Mutation	Protein Alteration
1(29)	25	male	sporadic	0.45	0.07	<10	0.25	delayed	anosmia	prelingual hypoacusis	absent	micropenis, cryptorchidism	c.122G>T	p.Gly41Val
2(29)	20	male	sporadic	–	–	–	–	delayed	anosmia	normal	absent	–	c.131C>G	p.Ala44Gly
3(29)	38	male	familial	0.2	0.12	27.29	0.3	delayed	anosmia	unilateral hypoacusis	absent	–	c.238C>G	p.Leu80Val
4(30)	19	female	sporadic	–	–	–	–	delayed	anosmia	prelingual hypoacusis	absent	primary amenorrhea	deletion exons 1-6	–
5(8)	30	male	sporadic	0.62	0.82	–	0.1037	delayed	anosmia	prelingual hypoacusis	depigmentation of the irides	hyperthyroidism, micropenis	c.565G>T	P.Glu189X
6(8)	21	male	–	0.4	0.25	–	2.43	delayed	anosmia	unilateral hypoacusis	hypopigmentation of the left iris	micropenis	–	p.Met108Thr
7(7)	15	male	sporadic	0.2	<0.7	–	–	delayed	anosmia	prelingual hypoacusis	depigmentation of the irides	cryptorchidism	c.434T>C	p.Leu145Pro
8(32)	31	male	–	–	–	–	–	delayed	anosmia	prelingual hypoacusis	absent	–	–	p.Met112Lle
9(33)	12	female	sporadic	1.0	2.6	<10	–	delayed	anosmia	prelingual hypoacusis	depigmentation of the irides, white hair	a lack of pubertal signs (breast and pubic hair, Tanner stage 1)	c.506delC	p.P169fsX117
10(34)	19	male	sporadic	0.54	0.15	–	<2.39	delayed	anosmia	prelingual hypoacusis	white hair	Bilaterally small testes (<3ml)	c.184G>T	p.Glu62X
11(9)	26	male	familial	0.2	0.4	–	<1.7	no	anosmia	unilateral hypoacusis	white hair	micropenis, cryptorchidism	c.2T>G	–
12(9)	18	female	sporadic	2.8	0.3	<10	–	no	anosmia	prelingual hypoacusis	absent	ptosis	c.331T>G	p.Phe111Val
13(9)	39	female	sporadic	<0.5	0.87	42	–	no	anosmia	prelingual hypoacusis	absent	obesity (BMI=51)	c.424T>C	p.Trp142Arg
14(9)	25	female	familial	2.1	0.8	20	–	delayed	anosmia	prelingual hypoacusis	absent	–	c.698-1G>C	–
15(9)	20	male	sporadic	–	–	–	–	delayed	anosmia	prelingual hypoacusis	absent	cryptorchidism	c.1290del	p.Ser431Argfs*71
16(9)	20	male	sporadic	–	–	–	–	–	anosmia	normal	absent	intellecual disability, dysmorphy, polymalformation	c.1298G>A	p.Arg433Gln
17(9)	33	male	familial? (a brother is most likely anosmic)	0.8	0.41	–	0.23	no	anosmia	prelingual hypoacusis	absent	ptosis	c.323T>C	p.Met108Thr
18(9)	19	female	sporadic	<1.0	0.5	<0.1	–	no	anosmia	normal	absent	macroscelia	c.451C>T	p.Arg151Cys

The age indicated was at the time of DNA sampling, usually age of diagnosis. Normal ranges are as follows: FSH/LH (2–20 UI/L); E2 (>20 pg/ml); and T (2–10 ng/ml). The following abbreviations are used: BMI, body mass index; E2, estradiol; T, testosterone; FSH, follicle-stimulating hormone; LH, luteinizing hormone.

The *SOX10* gene is located on chromosome 22q 13.1 and includes five exons, of which exons 3, 4, and 5 encode proteins. The SOX10 protein is a member of the 20 *SOX* gene family and contains 466 amino acids with a relative molecular mass of about 51kDa. The main functions are attributed to the highly conserved high mobility group (HMG) and the C-terminal transactivation domain (TAD) (17). The HMG box is composed of 80 amino acid residues (bits 102–181) and the stability of the whole structure is maintained by a hydrophobic inner core. It is able to specifically recognize and bind to the promoter DNA of the target gene, enabling the protein to act as a transcription factor (TF). When SOX10 protein binds to the target DNA, the DNA gap binds to the HMG box, resulting in a conformational change of the DNA, and the TAD architecture activates the transcription of the gene (18). Originally defined as a glial TF, SOX10 TF was found to be essential for the development of neural crest cells (NCC), such as olfactory ensheathing cells, melanocytes, intestinal ganglion cells, and many autonomic and sensory ganglion cells (19–21).

In 2013, Pingault et al. (9) found a mutation in the *SOX10* gene by performing a genetic test on a WS patient with an atrophied olfactory bulb whose clinical manifestations included sensorineural deafness and anosmia. It is speculated that *SOX10* mutation may lead to KS because KS patients with deafness have similar clinical manifestations to WS patients with anosmia. Pingault et al. (9) tested 17 KS patients who had been excluded from having known pathogenic gene mutations (e.g., *KAL1, FGFR1, FGF8, PROKR, PROK2, WDR11, HS6ST1, CHD7, SEMA3A*, etc.) for *SOX10* gene mutations and found seven *SOX10* mutations, six of which were pathogenic. The team then created a *SOX10* knockout mouse model and found that they exhibited a massive loss of olfactory ensheathing cells that hindered the migration signals of olfactory and GnRH neurons, causing characteristic clinical manifestations of KS such as anosmia and hypogonadism. Based on the above studies, a massive loss of olfactory ensheathing cells is considered as the main pathogenesis of KS patients with *SOX10* mutations.

The development and differentiation of NCC is a complex process regulated by multiple TFs and signaling pathways. *SOX10* plays an important role in this process and can regulate the development of NCC, especially melanocytes, either individually or synergistically with other TFs (22). In the pathogenesis of deafness in WS patients, studies (23, 24) have found that the potassium pump function of melanocytes in microphthalmia-associated transcription factor (MITF) mutant mouse models is abnormal and cannot maintain endolymphatic potential. Subsequently, no action potential was generated leading to secondary hair cell apoptosis. Moreover, *SOX10* can act on MITF and play its regulatory role in monomeric form or dimer form with *PAX3* (25, 26). As research continues, it has been reported that semicircular canal (SC) malformation is the most common malformation in WS patients with inner ear malformations. However, the phenotype of the inner ear malformation has not been linked to the genetic characteristics (27). In 2013, Elmaleh et al. (28) found bilateral SC malformations with vestibular and cochlear malformations along with *SOX10* mutations by genetic testing in 15 WS patients with inner ear malformations. Another study (29) has found that SOX10 protein is highly expressed during the early development of the inner ear and is consistently expressed throughout the development of the inner ear.

It has the function of maintaining the survival and differentiation of cochlear precursor cells, suggesting that there may be other pathways regulating the development of the inner ear in addition to the MITF pathway and in the mechanism of deafness caused by *SOX10* mutation. This pathway explains why WS patients with *SOX10* mutations are more prone to inner ear malformations, but relevant pathway has not been reported yet. In addition, SOX10 can also cooperate with MITF to activate dopachrome tautomerase (DTC) which acts in the biosynthesis of pigments. Therefore, *SOX10* mutation may cause abnormal pigmentation such as blue irises and white forehead hair (30, 31). In the nervous system, *SOX10* can directly regulate c-Ret which plays an important role in the development of the enteric nervous system (32). However, our patient did not suffer from neurological diseases (NIDs) such as Hirschsprung disease, which reflected the genetic and phenotypic heterogeneity. It has been proposed that this may be associated with *SOX10* haploinsufficiency (7). Consequently, the pathogenesis of WS is associated with the MITF pathway which is regulated by SOX10 along with other pathways that can regulate inner ear development, pigmentation, and nervous system.

A study found that the peripheral gonadal tissue of KS patients can still retain certain functions, and early hormone replacement therapy can increase testicular volume in some male patients and even produce sperm to gain fertility (33). In 2016, Maione et al. reported that a KS patient with *SOX10* mutation received testosterone injections in 1996 (age 21), 1999 (age 24), and 2000 (age 25). After treatment, the function of the hypothalamic-pituitary-gonadal axis returned to normal autonomously, and serum testosterone, gonadotropin, testicular volume, and sperm volume returned to normal as well. There was no recurrence for 20 years, but the olfactory bulb and SC remained hypoplastic (12). This was the first reported case till date of a KS patient with the *SOX10* mutation who recovered after treatment. Hence, it is essential to ensure the early and timely diagnosis and treatment of KS patients.

In conclusion, we identified a novel 20 bp insertion into the coding sequence of the exon4 of the *SOX10* gene causing a frameshift and denaturation of the *SOX10* protein of a patient with KS and WS2, suggesting that *SOX10* plays an important role in some critical stages of the development of neural crest cells. This supports previous data that *SOX10* mutations may be a common pathogenic factor for the development of both KS and WS. Therefore, *SOX10* mutation analysis should be considered for KS patients with combined WS clinical manifestations, especially deafness.

## Ethics Statement

The hospital ethics committee of China Medical University approved the study, and the patient and his family members provided written informed consent for publication of their clinical details and clinical images.

## Author Contributions

KC and YL designed the study. YL collected the data. KC drafted manuscript. YL and HW interpreted the datat and revised the manuscript. KC, HW, and YL approved the final version of the manuscript. All authors contributed to the article and approved the submitted version.

## Funding

This work was supported by the National Natural Science Foundation of China (grant no. 81300645).

## Conflict of Interest

The authors declare that the research was conducted in the absence of any commercial or financial relationships that could be construed as a potential conflict of interest.
